# *V*_*m*_-related extracellular potentials observed in red blood cells

**DOI:** 10.1038/s41598-021-98102-9

**Published:** 2021-09-30

**Authors:** Michael Pycraft Hughes, Emily J. Kruchek, Andrew D. Beale, Stephen J. Kitcatt, Sara Qureshi, Zachary P. Trott, Oriane Charbonnel, Paul A. Agbaje, Erin A. Henslee, Robert A. Dorey, Rebecca Lewis, Fatima H. Labeed

**Affiliations:** 1 Centre for Biomedical Engineering, University of Surrey, Guildford, Surrey, GU2 7XH UK; 2 School of Engineering, École Centrale de Lyon, 36 Avenue Guy de Collongue, 69134 Écully, France; 3 School of Veterinary Medicine, University of Surrey, Guildford, Surrey, GU2 7XH UK; 4 Present Address: MRC Laboratory for Molecular Biology, Francis Crick Avenue, Cambridge, CB2 0QH UK; 5 Present Address: Department of Engineering, Wake Forest University, 55 Vine St, Wake Downtown, Winston-Salem, NC 27109 USA

**Keywords:** Biophysics, Biomedical engineering

## Abstract

Even in nonexcitable cells, the membrane potential *V*_*m*_ is fundamental to cell function, with roles from ion channel regulation, development, to cancer metastasis. *V*_*m*_ arises from transmembrane ion concentration gradients; standard models assume homogeneous extracellular and intracellular ion concentrations, and that *V*_*m*_ only exists across the cell membrane and has no significance beyond it. Using red blood cells, we show that this is incorrect, or at least incomplete; *V*_*m*_ is detectable beyond the cell surface, and modulating *V*_*m*_ produces quantifiable and consistent changes in extracellular potential. Evidence strongly suggests this is due to capacitive coupling between *V*_*m*_ and the electrical double layer, rather than molecular transporters. We show that modulating *V*_*m*_ changes the extracellular ion composition, mimicking the behaviour if voltage-gated ion channels in non-excitable channels. We also observed *V*_*m*_-synchronised circadian rhythms in extracellular potential, with significant implications for cell–cell interactions and cardiovascular disease.

## Introduction

Since the work of Galvani^1^, it has been known that electricity plays a role in biological function. Subsequent work by Nernst^2^, Goldman^3^ and Hodgkin and Katz^4^ showed that the ionic imbalance between intracellular and extracellular spaces creates an electrochemical potential (termed the membrane potential *V*_*m*_) which plays a fundamental role in the function of muscle and nerves^5^. The standard expression for membrane potential is given by the Goldman-Hodgkin-Katz (GHK) equation, thus^3^:

1 $$V_{m} = \frac{RT}{F}ln\left( {\frac{{P_{{{\text{Na}}^{ + } }} \left[ {{\text{Na}}^{ + } } \right]_{out} + { }P_{{{\text{K}}^{ + } }} \left[ {{\text{K}}^{ + } } \right]_{out} + P_{{{\text{Ca}}^{2 + } }} \left[ {{\text{Ca}}^{2 + } } \right]_{out} { } + P_{{{\text{Cl}}^{ - } }} \left[ {{\text{Cl}}^{ - } } \right]_{in} { }}}{{P_{{{\text{Na}}^{ + } }} \left[ {{\text{Na}}^{ + } } \right]_{in} + { }P_{{{\text{K}}^{ + } }} \left[ {{\text{K}}^{ + } } \right]_{in} + P_{{{\text{Ca}}^{2 + } }} \left[ {{\text{Ca}}^{2 + } } \right]_{in} + { }P_{{{\text{Cl}}^{ - } }} \left[ {{\text{Cl}}^{ - } } \right]_{out} }}} \right)$$

where [*X*]_out_ and [*X*]_in_ are the extracellular and intracellular concentrations of the relevant ions, *P*_*X*_ are the permeability coefficients of the relevant ions, *R* and *F* the gas and Faraday constants, and *T* is the temperature. In non-excitable cells, *V*_*m*_ remains relatively constant (or “resting”), but the resting value varies considerably from one cell type to another^6^. Many mammalian cells exhibit a *V*_*m*_ about − 70 mV with respect to their surroundings, whilst red blood cells (RBCs) are nearer to − 10 mV. Voltages are maintained through membrane-based ion channels that regulate intracellular ion concentrations and transmembrane ion flows^7^. These in turn can be measured as resistances of the membrane and cytoplasm; changes in such properties have been shown to manifest during many cellular phenomena, including cancer metastasis^8^ and stem cell differentiation^9^. Recent work in our lab has shown that RBCs exhibit circadian rhythm variations in both membrane resistance and *V*_*m*_, driven by changing levels of cytoplasmic K^+^ and suggesting *V*_*m*_ may play a role in cellular clocks^10,11^.

**Figure 1:**
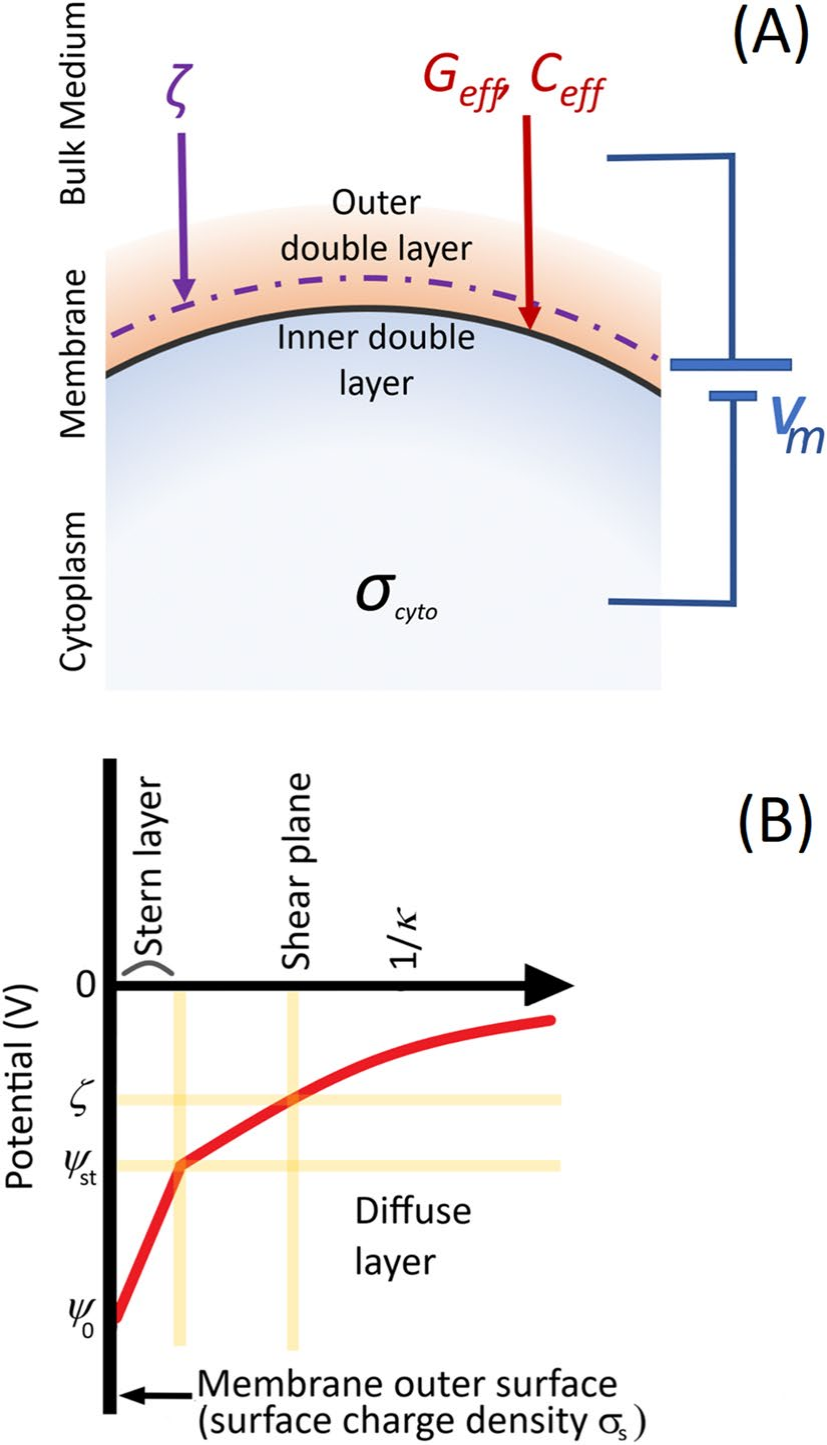
(**A**) A schematic of the cell, indicating the location of the electrical double layers, the shear plane (broken purple line) of *ζ*, the specific membrane conductance *G*_*eff*_ and capacitance *C*_*eff*_, cytoplasm conductivity *σ*_*cyto*_ and membrane potential *V*_*m*_. (**B**) The variation in potential from the cell surface, showing the location of the Stern layer and the shear plane at which *ζ* is located.

However, *V*_*m*_ is not the only electrical phenomenon which cells exhibit. The cell’s (typically negative) surface charge attract counterions, and repel coions or other negatively charged bodies in a region known as the electrical double layer, whose thickness is defined by the Debye screening length

2 $$1/\kappa = \sqrt {\left( {\frac{\varepsilon RT}{{2czF^{2} }}} \right)}$$

where *z* is the counterion valency and *c* the electrolyte concentration (mol m^−3^). The distribution of ions in the double layer is determined by the balance between electrostatic forces and thermal agitation, obtained by combining the Poisson equation with the Boltzmann distribution, leading to the Gouy-Chapman model^12^ which considers the electrical potential *ψ*(*r*) as a function of distance from the surface charge into an electrolyte, taking into account screening by counterions. Stern^13^ advanced model by adding a surface layer where the number density of ions ‘bound’ to the charged surface is unaffected by the ionic concentration of the electrolyte, whilst other ions the form a diffuse layer around the particle. The electrostatic potential falls exponentially from the potential at the end of the Stern layer *ψ*_St_; the electrical potential at distance *r* from the surface is given by

3 $$\psi \left( r \right) = \psi_{{{\text{St}}}} e^{ - (\kappa r)} ,$$

as shown in Fig. 1. The presence of the countercharge in the double layer immediately outside the cell membrane makes direct measurement of the surface charge difficult; however, its influence can be measured in the electrical potential *ψ*(*r*) at the hydrodynamic plane of shear (ca. 1 nm outside the membrane), which marks the plane inside which the ions which act as if attached to the particle. The voltage at the shear plane is called the *ζ*-potential^12,14^. In theory, *ζ* is a product of cellular surface chemistry (principally sialic acid residues) and double layer thickness. *ζ*-potentials have been studied in a number of cells, particularly in RBCs where changes in *ζ* have been associated with the formation of rouleaux and changes in the erythrocyte sedimentation rate ESR^15,16^. It is also known that RBCs stored for long periods are known to exhibit a reduction in ζ over time^17^.

Since *V*_*m*_ is dependent on ion transport through the membrane, there is a strong case that the electrical double layer plays a role in determining *V*_*m*_. As is clear from the Gouy-Chapman model, the ion concentration at the cell surface is equal not the concentration in the bulk, as used in the GHK equation. Instead, the ion concentration at the surface is described by the Poisson–Boltzmann equation^12^:

4 $$c_{i} \left( 0 \right) = c_{oi} exp\left( {\frac{{ - z_{i} e\varPsi }}{kT}} \right)$$

where *c*_*i*_(0) is the concentration of ion *i* at the Stern layer, *c*_*oi*_ is the concentration of the ion in the bulk, *z* is the valency, and *e* is the charge. Ions carrying the same charge as the cell surface (coions) are electrostatically repelled by charges of like sign, resulting in diminishing coion concentration near the surface; conversely, the counterions increase in concentration near the surface by electrostatic attraction. Such factors are typically considered to be part of the permeability coefficients of the GHK equation. However, the reverse is not taken to be true; the membrane potential affects does not affect the extracellular ion concentration. The effect is taken to be causal in entirely one direction.

We examined *V*_*m*_ and *ζ* of red blood cells (RBCs) subject to fifteen combinations of ionic strength and surface treatments intended to modify either *V*_*m*_ or *ζ*, and observed transient changes in both parameters on both short (minute) and long (daily) timescales. We report that whilst *V*_*m*_ is affected by *ζ*, *V*_*m*_ also alters *ζ* by up to 37% of *V*_*m*_ via capacitive coupling between the cell interior and the bulk medium. Furthermore, the best-fit model to behaviour also accurately describes observed behaviour attributed to RBCs, including mimicking the behaviour of voltage-activated ion channels in patch-clamp experiments, for which no such channels have been identified.

## Results

### *ζ* exhibits *V*_*m*_-dependence in RBCs

**Figure 2:**
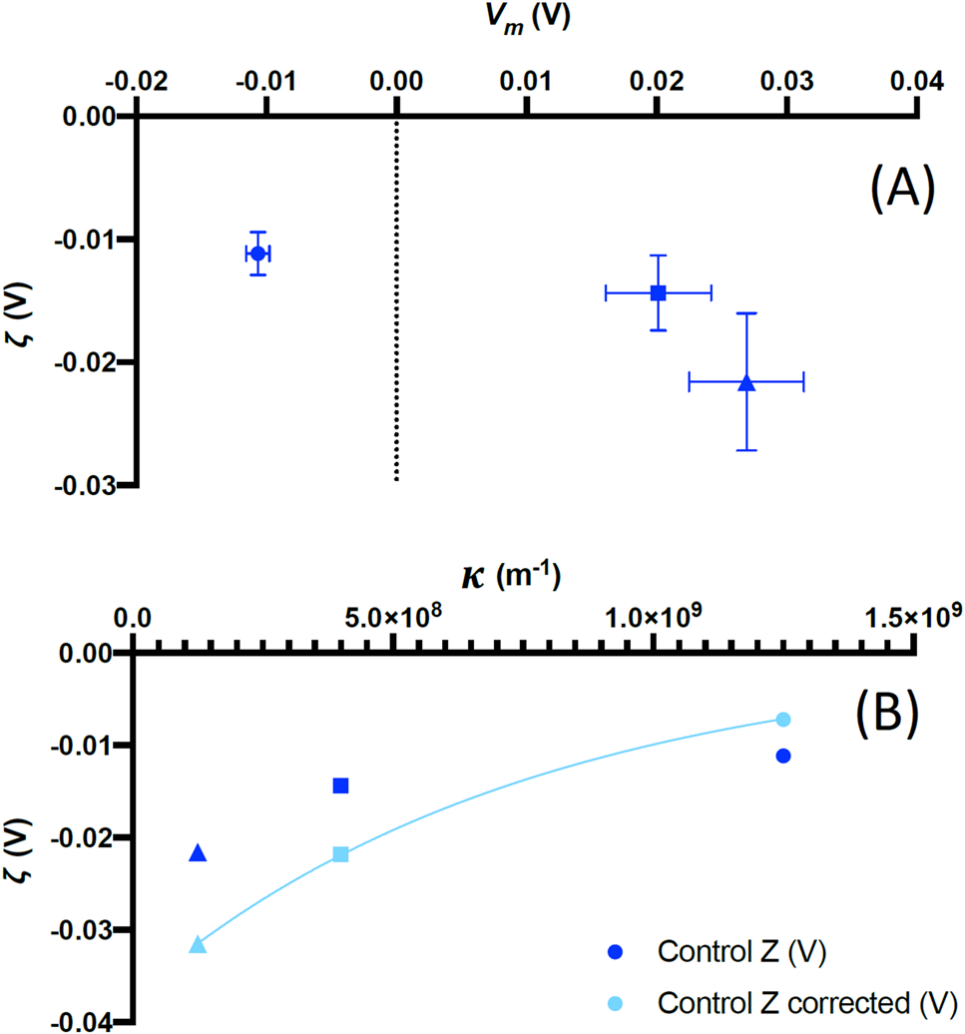
(**A**) The mean variation (± s.d.) in *V*_*m*_ and *ζ* for control RBCs in media containing 100% (square), 10% (circle) and 1% (triangle) physiological saline as described in the text. (**B**) The same values of *ζ* plotted against the calculated reciprocal Debye length *κ* for each medium, together with values adjusted by 0.37 *V*_*m*_. The relationship between these variables theoretically follows a negative exponential to which the adjusted values offer a perfect (r^2^ = 1) fit. All points represent the average of four donors.

We first considered untreated RBCs, which we examined in three isosmotic media with different ionic strengths (referred to here as 100%, 10% and 1% according to the proportion of saline in the medium, with the remainder comprising iso-osmotic sugar solution (see Methods for details). Lowering ionic strength is known to change *V*_*m*_ in RBCs^18,19^, but also changes the thickness of the double layer which is dependent on ionic concentration (see Eq. (2)). Cells equilibrated for 30 min before measurements commenced; the relationship between *V*_*m*_ and *ζ* is shown in Fig. 2a*.* Equation (3) suggests that *ζ* should depend on 1/*κ* with *ψ*_St_ constant. However, when we plotted measured values of *ζ* against calculated values of *κ* from Eq. (2) and attempted to fit Eq. (3) to these points, we found the fit was greatly improved through the addition of a term proportional to *V*_*m*_ (as shown in Fig. 2b). The best fit was obtained by calculating ζ from Eq. (3) plus an additional term proportional to *V*_*m*_ and independent of double-layer thickness the ion concentration in the medium. When we included a coefficient of proportionality, *Ξ*, between *V*_*m*_ and the change in *ζ*, the best fit is achieved when*:*

5 $$\begin{aligned} \zeta & = \psi_{{{\text{St}}}} e^{ - (\kappa x)} + \varXi V_{m} \\ & = \zeta^{^{\prime}} + \varXi \, V_{m} \\ \end{aligned}$$

where ζ′ is the component of the *ζ*-potential due *only* to the surface charge. When *ΞV*_*m*_ was included, the fit for *ζ* and *κ* improved significantly, with a perfect fit (r^2^ = 1) between Eq. (4) and the data achieved when *Ξ* = 0.37, *ψ*_St_ =  − 0.037 mV, and the shear plane is 1.0 nm from the end of the Stern layer.

**Figure 3:**
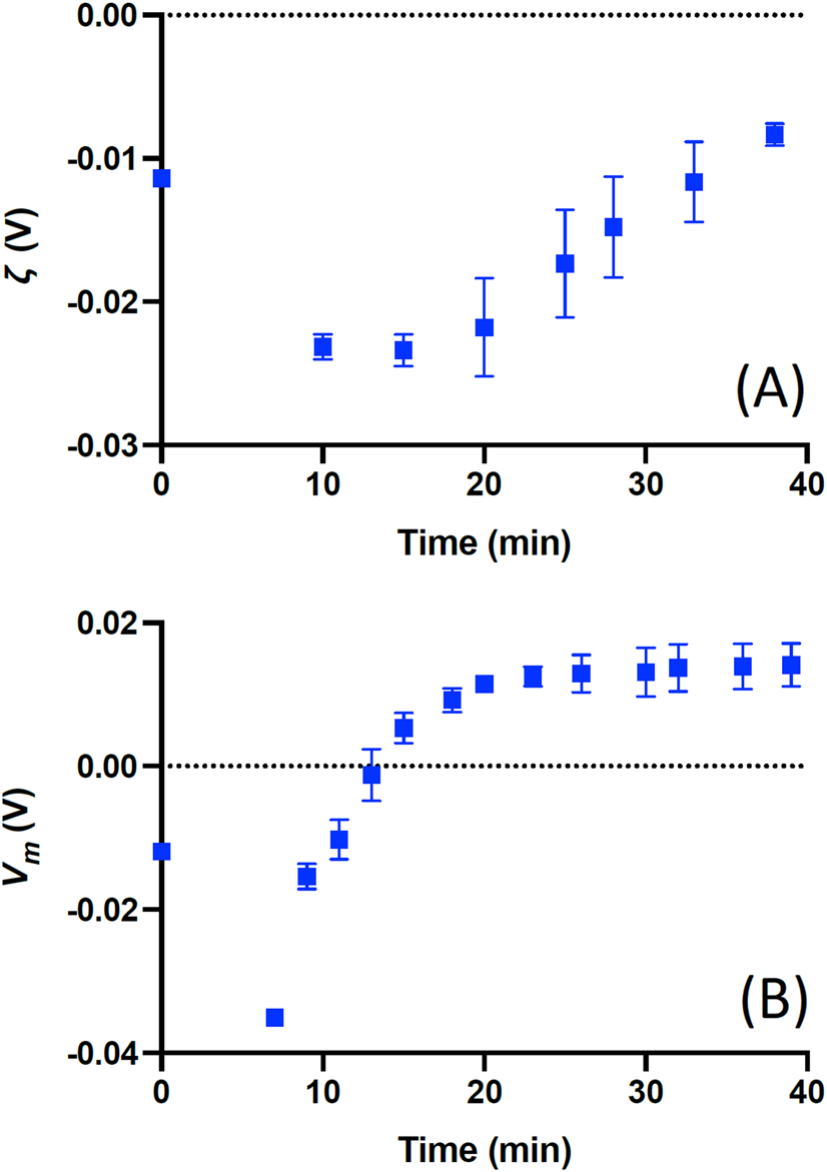
The mean (*n* = 3) *V*_*m*_ (**A**) and *ζ*-potential (**B**) of RMCE immediately after resuspension into 10% solution (see text). As can be seen, in both instances the value immediately changes from a rest value (taken in 100% solution, denoted here as time 0), peaks and then increases to a rest value. The transition period was consistent for both *V*_*m*_ and *ζ*, but the former was observed to respond consistently across all three samples, whereas for *ζ* a delay of 5–15 min was observed before transition began.

We extended this observation by observing dynamic changes in both *V*_*m*_ and *ζ*. It is known^18,19^ that the *V*_*m*_ of RBCs changes rapidly immediately after resuspension in low ionic strength solution, before reaching steady state some 30 min later. We investigated whether this could be observed in both *V*_*m*_ and *ζ* by measuring at ≤ 5 min intervals after resuspension in 10% medium. The results (Fig. 3) show similar peaks in both *V*_*m*_ and *ζ* immediately after resuspension, which then transited to a new steady-state value over approximately 20 min. Interestingly, whilst changes in *V*_*m*_ showed little variation in timing across the samples, measurements of *ζ* exhibited a pause of between 5–15 min before beginning equilibration. However, once the equilibration in *ζ* began, it took place at the same rate as measurements in *V*_*m*_; once this delay was taken into consideration, comparison of measured values of *V*_*m*_ and *ζ* suggests *Ξ* = 0.35 across all samples.

### Alteration of the membrane alters the relationship between ζ and *V*_*m*_

**Figure 4:**
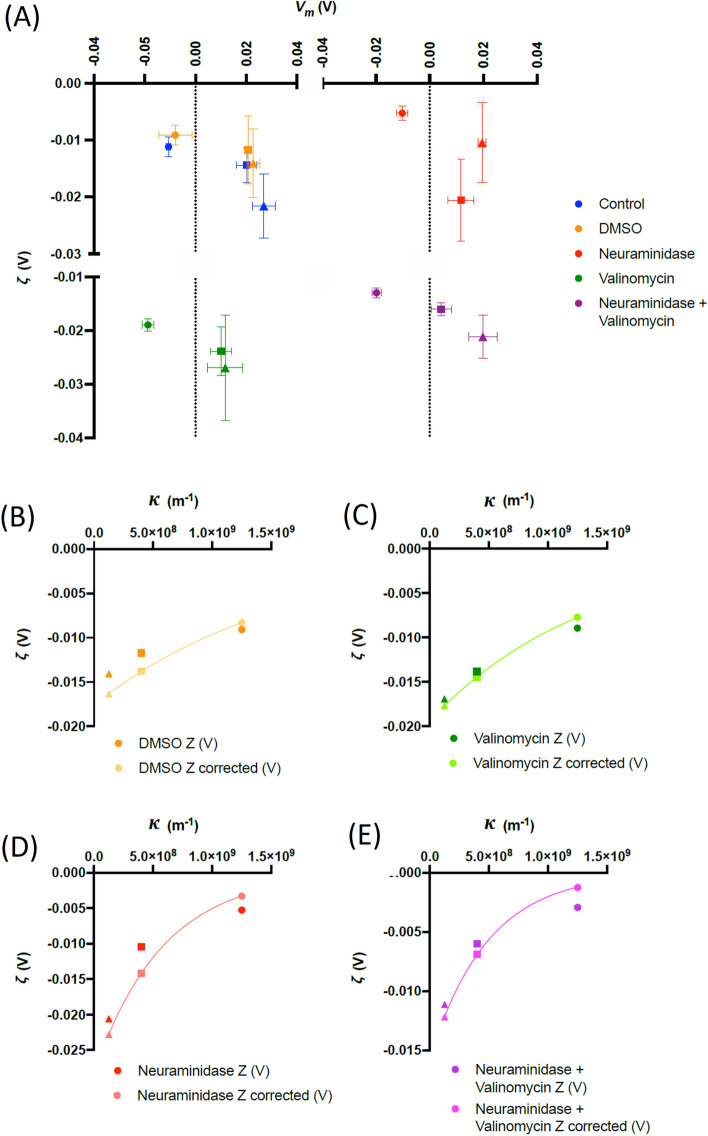
(**A**) The mean variation (± s.d.) in *V*_*m*_ and ζ for RBCs suspended in media containing 100% (square), 10% (circle) and 1% (triangle) physiological saline as described in the text. RBCs were treated with valinomycin, neuraminidase, and a combination of both, as well as a DMSO was control. All points represent the average of four donors. (**B**–**E**) The values of *ζ* from Fig. 4A plotted against the reciprocal Debye length *κ*, together with values adjusted by *ΞV*_*m*_ as described in the text. The relationship between these variables theoretically follows a negative exponential, to which the adjusted values offer a perfect (R^2^ = 1) fit. All points represent the average of four donors.

We then repeated the experiment with cells treated using chemical agents to alter the membrane properties. Cells were treated with valinomycin (previously demonstrated to hyperpolarize RBCs)^10^, neuraminidase (which removes charge groups from sialic acids on the membrane surface)^20^ and a combination of both. Finally, a vehicle control (DMSO, used to dissolve valinomycin) was also studied. When cells were suspended in 100% saline solution, we found that neuraminidase lowered *ζ* with respect to untreated cells, as expected*.* However, valinomycin (which alters *V*_*m*_ but does not alter the membrane surface charge) altered both *V*_*m*_ and *ζ*. Combining neuraminidase and valinomycin produced an additive effect of the two treatments administered separately; when cells were suspended in isosmotic media of different ionic strength, a similar trend was observed (Fig. 4) to that seen for untreated RBCs. As before, superior fits were observed using Eq. (5) than Eq. (3). Neuraminidase fitted (r^2^ = 1) with *Ξ* = 0.19; where DMSO was used (i.e. treatments of valinomycin, valinomycin + neuraminidase, or vehicle control), optimal fits (r^2^ = 1) were achieved when *Ξ* = 0.085 ± 0.018. Estimated values of *ψ*_St,_ the location of the shear plane *x* and the value of *Ξ* used can be seen in Table 1.

**Table 1:** Estimated electrical properties for RBCs.

	*Ψ*_St_ (V)	*X* (nm)	*Ξ*	*C*_*shear,*_ Fm^−2^	*Ψ*_St_ Ratio	*C*_*shear*_ Ratio
Control	− 0.037	1.0	0.37	0.232	1	1
+ Valinomycin	− 0.020	0.7	0.07	0.123	0.54	0.53
+ Neuraminidase	− 0.028	2.0	0.19	0.175	0.76	0.75
+ Valinomycin + Neuraminidase	− 0.016	2.0	0.09	0.100	0.43	0.43
DMSO Control	− 0.018	0.6	0.1	0.125	0.49	0.54

**Figure 5:**
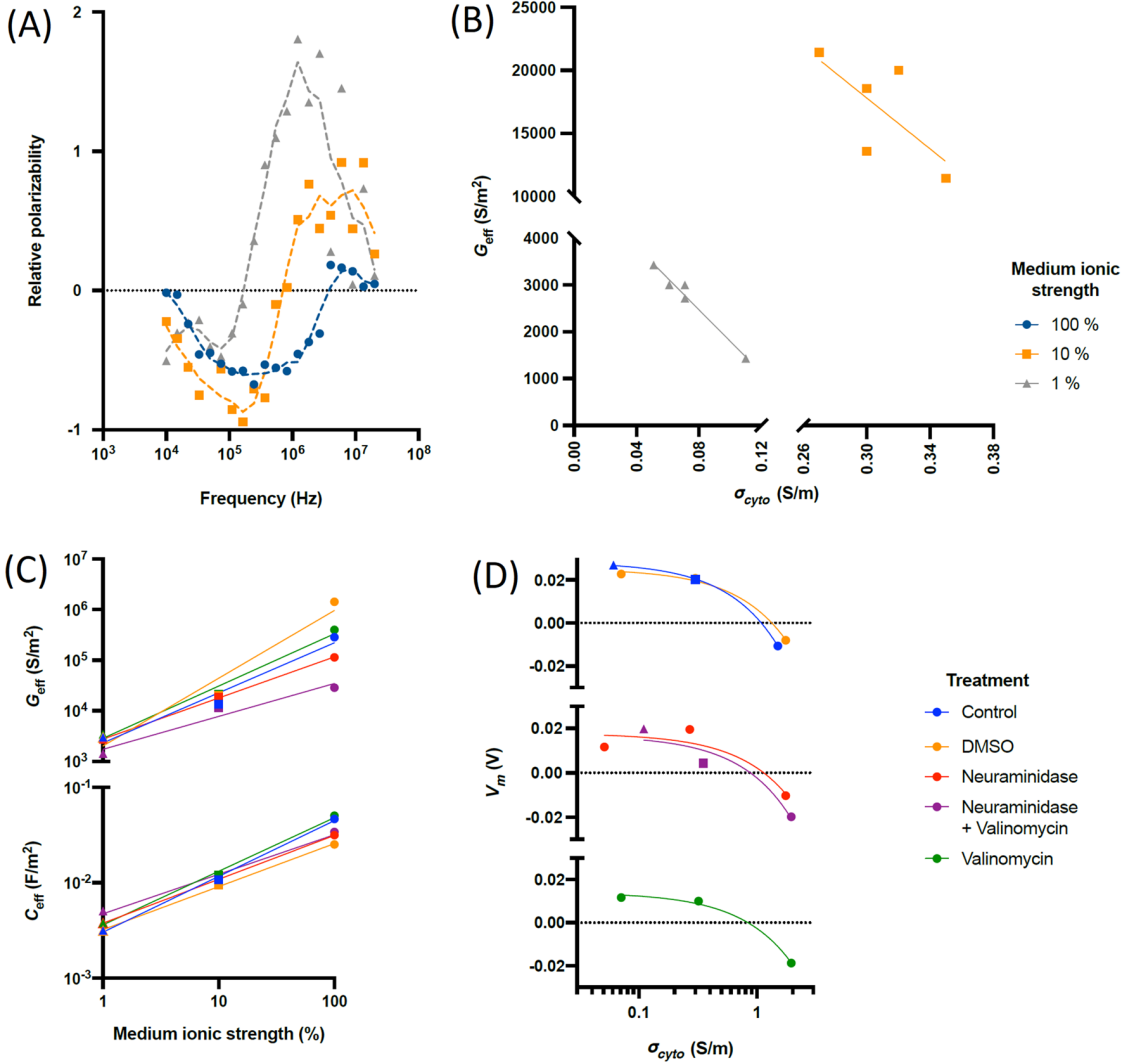
(**A**) The DEP spectra (points) of RBCs in media of different ionic strength (1% (grey), 10% (orange) and 100% (blue)) together with rolling average trendlines; (**B**) the values of *G*_*eff*_ vs. *σ*_*cyto*_ for 1% and 10% media, together with best-fit trendline; (**C**) Specific membrane conductance *G*_*eff*_ and capacitance *C*_*eff*_ as a function of medium ionic strength; (**D**) the relationship between cytoplasm conductivity *σ*_*cyto*_ and membrane potential *V*_*m*_.

The estimated values of surface potential *Ψ*_St_ and shear plane thickness from the best fit curves shown in Figs. 2 and 5 after adjusting *ζ* by an amount *Ξ* multipled by the measured *V*_*m*_. When the capacitance between shear plane and membrane *C*_*shear*_ was calculated, the *Ψ*_St_ ratios and *C*_*shear*_ ratios show a high degree of similarity.

### The relationship between *ζ* and *V*_*m*_ is unaffected by double layer thickness or medium composition

We repeated this procedure using untreated RBCs suspended in four isosmotic solutions of different ionic composition, changing both *V*_*m*_ and the thickness of the double layer 1/*κ*. The solutions used were: (i) 145 mM KCl; (ii) 145 mM NaCl; (iii) 72.5 mM KCl and 72.5 mM NaCl; and (iv) 97 mM CaCl_2_. We compared the value of *ζ* measured by electrophoretic mobility with *V*_*m*_ calculated using the GHK equation (Eq. (1)) inserted into Eq. (5) to investigate the relationship between *ζ* and *ψ*_St_. We found that the best fit was achieved when *Ξ* = 0.27; assuming *x* = 0.7 nm as before. This yielded estimates of *ψ*_St_ = -30.0 ± 2.5 mV for all four cases (Table 2), similar to the values determined in the first study. That this holds for all four cases is notable, as whilst (from Eq. (2)) the double layer is substantially thinner for CaCl_2_ due to the divalent ion Ca^2+^, *Ξ* remains the same, suggesting *Ξ* is independent of double-layer thickness.

**Table 2:** Estimated electrical properties of RBCs in different ionic media.

	Measured *ζ* (V)	Estimated *V*_*m*_ from GHK (V)	*V*_*m*_ (V)	Estimated *ψ*_St_ (V)
NaCl	− 0.0091 ± 0.0027	− 0.014	0.0058	− 0.034
KCl	− 0.0108 ± 0.0012	− 0.001	0.0008	− 0.028
NaCl/KCl (50:50)	− 0.0090 ± 0.0021	− 0.008	0.0030	− 0.028
CaCl2	− 0.0027 ± 0.0026	− 0.060	0.0088	− 0.030

The measured value of *ζ* in different ionic media, together with the calculated value of *V*_*m*_ for each medium determined using the GHK equation. We used the calculated value of *V*_*m*_ to determine a *Ξ* = 0.27, and determine *ζ’* using Eq. (5). This was used in Eq. (4) to estimate *ψ*_St_ due solely to the surface charge, which showed a high degree of similarity across the four cases.

### *V*_*m*_-dependence also affects the passive electrical properties

We also examined the passive electrical properties of effective membrane conductance *G*_*eff*_ and capacitance *C*_*eff,*_ and cytoplasm conductivity *σ*_*cyto*_ of the cells in 100%, 10% and 1% solutions using DEP. We empirically analysed the results to find relationships of note, by examining RBC response as a function of different medium conductivity, and as a function of chemical modification. Key findings are shown in Fig. 5. When we examined RBCs at common medium ion concentrations, we found strong negative linear correlations between *σ*_*cyto*_ and *G*_*eff*_. This was strongest (r = 0.97) in lowest conductivity media, with the data for *G*_*eff*_ becoming noisier at higher conductivities due to the emergence of an additional low-frequency polarisation in the low-frequency band where *G*_*eff*_ dominates (discussed below). This negative correlation tallies with observations reported by Henslee et al.^10^, where *σ*_*cyto*_ and *G*_*eff*_ were observed to vary in antiphase through the day, and suggests that these two parameters have a common origin. We also found a strong dependence between medium conductivity, *G*_*eff*_ and *C*_*eff*_. This has been reported in limited fashion for RBCs previously^21^ but is not observed in other cells, such as platelets or Jurkats^22^. We found *C*_*eff*_ could be modelled empirically to within ± 15% as an additional capacitive layer beyond the membrane with width equal to the double layer thickness*.* We also observed that *G*_*eff*_ varied approximately linearly with ionic strength, indicating a dependence both to this and *σ*_*cyto*_. We found a very significant linear relationship (r^2^ > 0.99) between *V*_*m*_ and *σ*_*cyto*_ for all cases except cells treated with neuraminidase (where r^2^ = 0.86), of the form *V*_*m*_ = *A* − *B.σ*_*cyto*_. We found that *A* was 85 ± 10% higher in cells without valinomycin than with, whilst *B* was 47 ± 6% higher in control cells than those in DMSO, suggesting *V*_*m*_ is affected by a change in K^+^ transport (in line with Eq. (1)), and DMSO affects how *V*_*m*_ is affected by cytoplasm composition.

**Figure 6:**
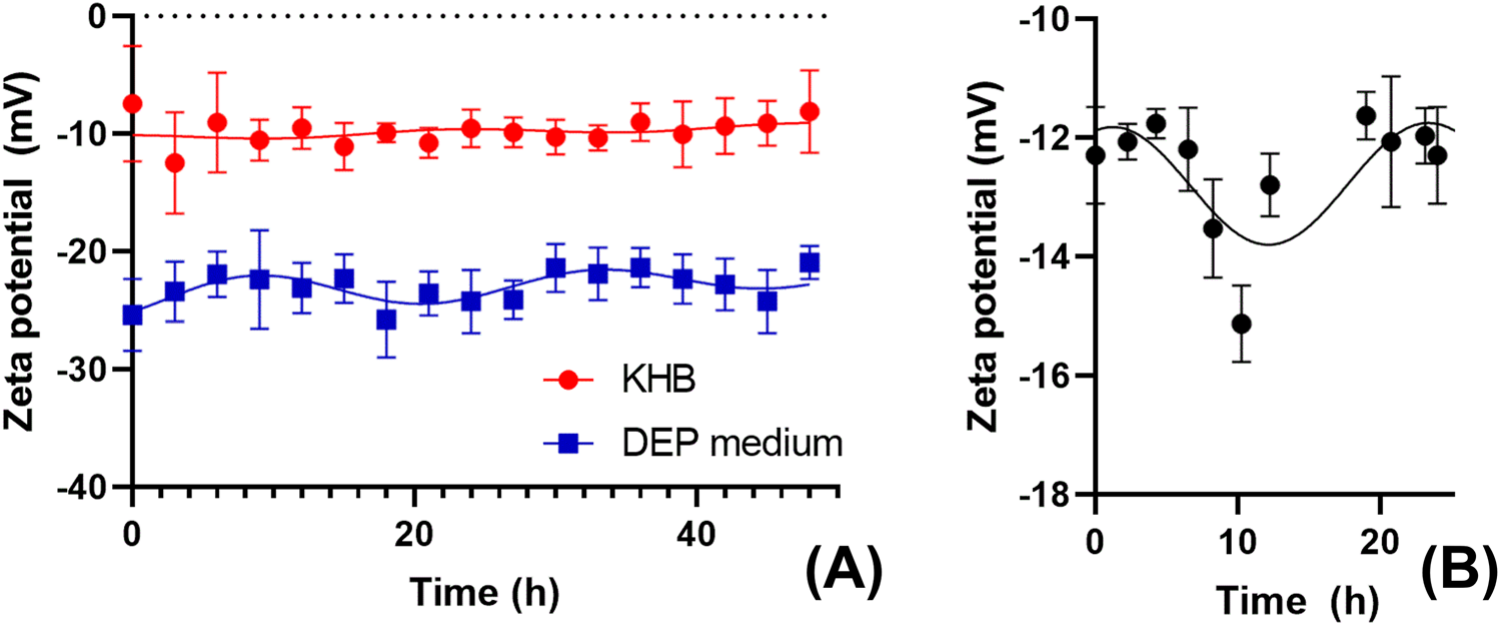
Circadian behaviour of *ζ*-potential in RBCs. (**A**) mean (n = 4) *ζ* for RBCs entrained and suspended in KHB and DEP medium, together with best-fit rhythm with period of 24.5 h and 24.3 h respectively. (**B**) *ζ* of RBCs taken directly from a participant over a 24 h period, measured within 60 s of donation, together with a best-fit rhythm with 22.2 h period (time 0 h corresponds to 10am).

Whilst DEP behaviour generally followed the classical models for a shelled spheroid, we observed an increase in polarizability at low frequencies (Fig. 6) similar to that observed in nanoparticles, and attributed to polarisation of the electrical double layer^23^. That paper suggested that the frequency below which the effect occurs depends on a time constant τ. We found that amending the standard model to incorporate this exactly matched our observations. Prior work^23^ suggests that τ is inversely proportional to surface conductance *k*_*s*_*.* Furthermore, we found a linear relationship (fitted with r^2^ > 0.99 in all cases) between 1/ τ and *ζ’,* the *ζ*-potential component due *only* to the surface charge, which followed a linear form of *k*_*s*_ = *P.ζ’* + *Q,* with *P* being 8.9 ± 1.6 × larger in the presence of DMSO than without, and *Q* being 4.8 ± 1 × larger. This suggests that the low-frequency rise is indeed due to double layer polarisation, and is strongly affected by double layer composition but is unaffected by *V*_*m*_; it may also suggest that the effect is influenced by both the diffuse layer conduction^24^ in term *P* and Stern layer conduction^23^ in term *Q*.

### ζ exhibits an endogenous circadian rhythm

We also examined the variation in ζ over a 48 h period, in comparable conditions to experiments measuring *V*_*m*_ and DEP parameters taken by Henslee et al.^10^ Cells in both media demonstrated rhythms (Fig. 6a) when fitted a damped cosinor curve, with periods for of 24 h 53 min (KHB) and 24 h 17 min (DEP medium). The mean values of *ζ* in were -11.4 mV (KHB)and -24.0 mV (DEP medium). The amplitude of the rhythm in DEP medium was larger at 1.8 mV compared to 0.25 mV in KHB, and the signal was also almost exactly in antiphase (phase difference of 10 h 30 min); this can be explained by the change in *V*_*m*_ polarity between low- and high-ionic strength media, as described above. The DEP medium rhythm was statistically preferred to a straight line (*p* < 0.0001), though the KHB rhythm was not.

When we examined blood immediately after donation over a 24 h period, we found that *ζ* again preferentially fitted a damped cosinor (*p* > 0.0001) with a 22 h 9 min period, a -12.9 mV baseline and 1.0 mV amplitude.

If we compare the amplitude of the *ζ* rhythm in DEP medium with the published data on *V*_*m*_ rhythms by Henslee et al.^10^, it suggests a value of *Ξ* of approximately 0.2; this is lower than calculated for other RBC conditions, though this may be an underestimate due to the relatively low time-precision of the *V*_*m*_ data, or indicative of a reduction in *Ξ* over the 48 h experimental time after entrainment.

## Discussion

### The relationship between *V*_*m*_ and *ζ* can be observed in cells exhibiting voltage-dependent ion channel behaviour without voltage-dependent channels

If there is a causal relationship between *V*_*m*_ and ζ, such that altering *V*_*m*_ alters ζ, which would then have a concomitant effect on *V*_*m*_, then it raises significant questions about our understanding of *V*_*m*_ and of the validity of the GHK equation. Equation (1) states that *V*_*m*_ is solely dependent on intracellular and extracellular ion concentrations, but not on the current value of *V*_*m*_; this may need to be adapted to include a feedback component to account for changes in extracellular ion concentration due to *V*_*m*_.

Evidence for such a relationship may be found in previous electrophysiological studies, where *V*_*m*_ is known and deliberately altered and changes in behaviour observed. For example, the relationship between *V*_*m*_ and *ζ* provides an explanations for RBC’s voltage-dependent, non-specific cation channel, whose function has been observed in patch-clamp experiments^25–27^ but whose action has never been correlated with a particular ion transporter. The behaviour of this putative channel is characterised by a cation efflux at holding potentials greater than + 20 mV.

**Figure 7:**
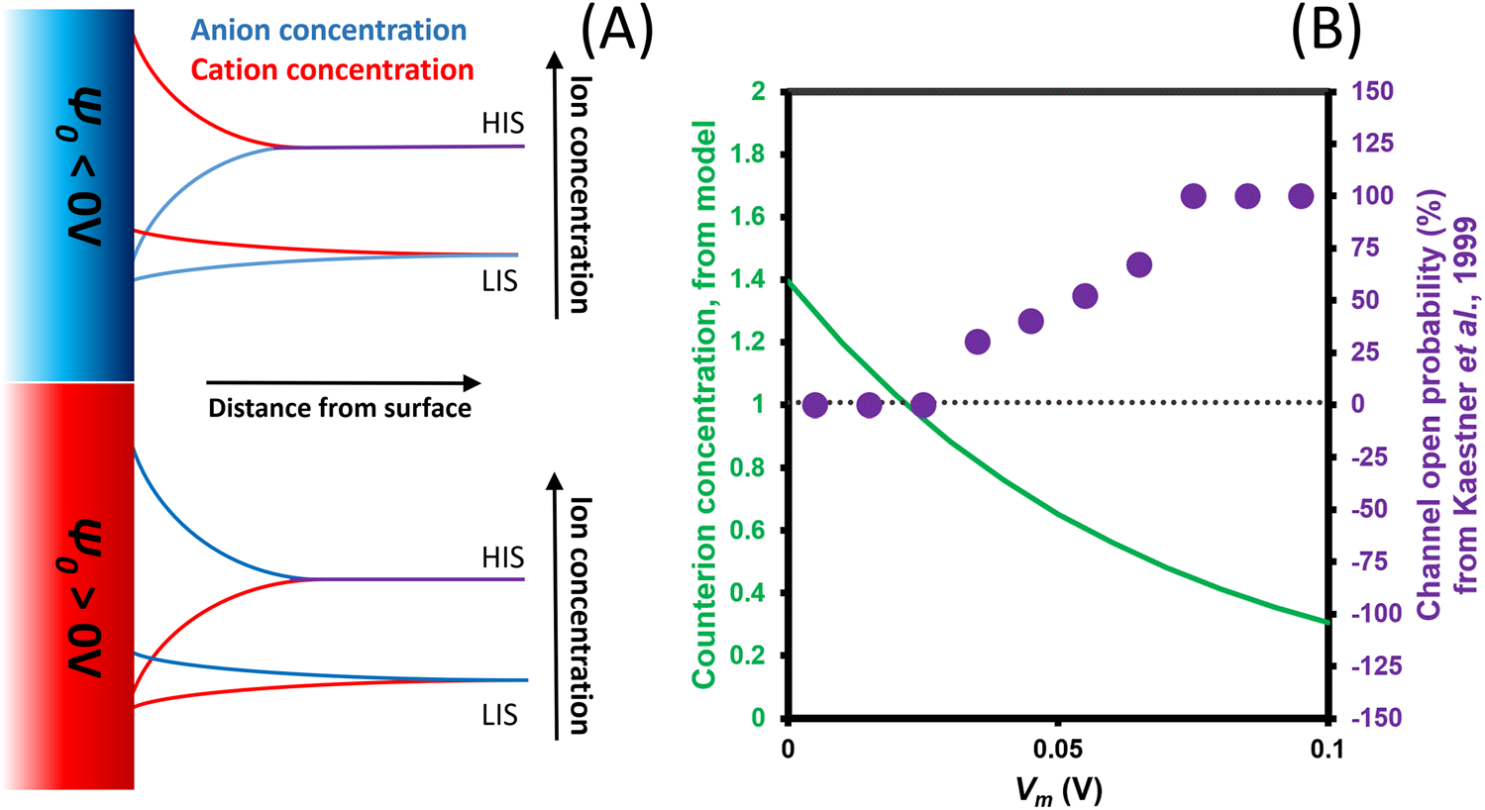
(**A**) A schematic showing ion concentrations in proximity to the cell surface in high ionic strength (HIS) and low ionic strength (LIS) solutions. Where the surface potential of a cell is negative, it will electrostatically attract cations from solution, raising the cation concentration at the surface whilst depleting anions. Where the surface potential is positive, this situation is reversed, with the cation level in HIS solutions reaching similar values to those observed in LIS solutions. (**B**) A comparison of the voltage-gated cation channel behaviour reported by Kaestner et al.^26^, and the cation concentration at the surface relative to the bulk (green line) determined using the Poisson-Boltzmann equation, using *ζ* determined from Eq. (5).

It is also known that RBCs exhibit significant potassium efflux when resuspended in media of low ionic strength^19^. It is important to note that the ion concentration at the cell surface is equal not the concentration in the bulk, as used in the GHK equation, but is instead described by the Poisson-Boltzmann equation (Eq. (4)). This describes diminishing coion concentration and increasing counterion concentration near the surface, as shown schematically in Fig. 7A for positive and negative values of *ψ*; as can be seen, the high and low ionic strength coion concentrations are similar.

If we replace *ψ* in Eq. (4) with *ζ* as determined from Eq. (5) with *Ξ* = 0.37 and *ζ’* = -0.072 V, we can determine the ion concentration at the shear plane and hence, available for membrane-based ion transporters. Figure 7B shows the cation concentration at the cell surface relative to the bulk. Below *V*_*m*_ = + 19.5 mV we find cation concentration at the surface is higher than the bulk (whilst anion concentration would be lower); as *V*_*m*_ is elevated beyond this, the cation concentration at the surface drops below that in the bulk, potentially sufficient to shift the cell to elevated ion leakage behaviour as observed in low ionic strength media. Significantly, there is a striking correlation between the surface cation concentration and the “% open probability” for the putative nonspecific voltage-activated cation channel reported by Kaestner et al.^26^ with 100% probability of channel opening coinciding with an external cation concentration of approximately 50% that of bulk medium.

We propose that rather than indicating the presence of a voltage-activated ion channel, the effect is due to *V*_*m*_ altering *ζ* and hence the ion availability for transport. Furthermore, since changes in the ion permeability due to the low external concentration would cause concomitant elevation in *V*_*m*_ (from the GHK equation), this may make the rise of *V*_*m*_ self-sustaining whilst ion transfer occurs, explaining the hysteresis effect observed in patch-clamp studies of the putative channel^28^. This result is highly significant as it suggests a mechanism describing observed RBC behaviour without requiring any voltage-dependent molecular mechanisms. If the same effect is observed more generally in other cell types, this has significance for our understanding of voltage-activated channels, and also of the GHK equation, dependent as it is on bulk, rather than surface, ion concentrations.

### The mechanism of transmission of *V*_*m*_

The data suggest that electrical properties such as *ζ*, *V*_*m*_, *G*_*eff*_, *C*_*eff*_ and *σ*_*cyto*_ form an interconnected cellular electrome; and that an electric field due to *V*_*m*_ extends beyond the membrane to at least the plane of shear. We can use the data generated from modelling in order to elucidate the physical principles underpinning *Ξ*.

Classical charged particle theory assumes a solid particle possessing a surface charge. However, a cell is not solid, but rather a membrane with surface charges on both outer and inner faces surrounding a charged core, where the electric field distribution away from the cell is a function of both the surface charge on the outer surface and the surface charge on the inner surface^29^. An obvious potential mechanism for relating *V*_*m*_ to *ζ* would be if the membrane potential, being dropped across the capacitive membrane, produced charges on the surface in accordance with the principle *Q* = *CV*; this would then change the surface charge and lead to an altered value of *ψ*_0_ and hence *ζ*. However this model fails to replicate observed behaviour, since the change in *ζ* would vary according to the value of 1/*κ*, and hence with medium ionic strength. This is quite different to our observations, where the additional term *ΞV*_*m*_ appears to be independent of the double layer thickness.

Examination of *Ξ* shows that values fall broadly into two categories; untreated cells and those treated with neuraminidase have higher values of *Ξ* (0.19–0.37), whilst cells treated with DMSO or treatments requiring DMSO for resuspension exhibit values of *Ξ* of approximately 0.08. Cells treated with DMSO also demonstrated lower values of *ζ* than control, although no mechanism is reported for it altering surface charge. Table 1 also suggests *ψ*_0_ is higher for non-DMSO conditions (control, neuraminidase) than DMSO-treated cells, and differences in behaviour for DMSO-treated vs untreated cells were also observed in the relationship between *V*_*m*_ and *σ*_*cyto*_. DMSO has been reported to cause membrane thinning, and increases membrane fluidity and permeability^30^ displacing water at the membrane surface^31^, and has a significantly lower relative permittivity than water^32^. Recent examination of the double-layer capacitance surrounding electrodes^33,34^ has shown that solution permittivity exhibits a much greater influence on double-layer capacitance than ion concentration, and that the double-layer specific capacitance of DMSO is only 0.14 Fm^−2^ compared with 0.35 Fm^−2^ for water, due to the Stern layer being wider due to the relative size of the solvent molecules (0.37 nm for DMSO, 0.16 nm for water) rather than differences in permittivity (5.7ε_0_ for DMSO, 6.3ε_0_ for water).

We calculated the total capacitance between cell surface and slip plane *C*_*shear*_ by assuming a series combination of Stern layer capacitance as described above (assuming that DMSO displaced water at the interface, where used), and a shear plane capacitance calculated using the permittivity of water (78ε_0_) and the shear plane thicknesses derived in Table 1. Remarkably, when we compared the inter-condition ratios between the calculated values of capacitance, with the ratios of the estimated values of *ψ*_st_ we found an exceptionally high degree of correlation (Table 1). We suggest that this strongly validates both the model of capacitance between membrane and shear plane.

Furthermore, when we developed empirical models to elucidate physical principles underlining *Ξ*, we found we were able to obtain excellent fits using the equation:

6 $$\varXi = \frac{{\alpha C_{shear}^{2} }}{{1 + C_{shear}^{2} }}$$

where *α* is a dimensionless constant of value 6.7. This produced values of *Ξ* of 0.34 for control cells, 0.19 for neuraminidase-treated cells, and 0.065–0.10 for the three DMSO treatment conditions. Interestingly, The dependence on the *square* of the capacitance is echoed in the relationship between potential and capacitance in semiconductor physics of PN junctions, in which a potential is applied across a barrier comprising different concentrations of positive and negative charge carriers, suggesting that this may be a route to a more analytical model of *Ξ*.

### The physiological implications of *ζ* dependence on *V*_*m*_

The modification of *ζ* by *V*_*m*_ may explain phenomena such as the “storage lesion” in blood-banked RBCs, where storage results in cells reducing in *ζ*-potential without a commensurate loss of sialic acid from the membrane^17,35^, may be due to loss of cytosolic K^+^ in the days following storage^36^, causing a depolarization of *V*_*m*_ leading to a reduction loss in *ζ*. *ζ* has also been implicated in the formation of rouleaux^15,16^; our results suggest that changing either the permittivity of the double layer or the value of *V*_*m*_ may modulate this behaviour. This is consistent with observations that the erythrocyte sedimentation rate (ESR) of whole blood is modulated by K^+^ channel blockers such as quinine^37^.

Whilst we observed changes in *ζ* in both entrained and ex vivo samples, there are no reports in the literature of circadian behaviour in blood sample measurements of *ζ*, or of the most common clinical measurement in which *ζ* takes a role, that of erythrocyte sedimentation rate. One possible reason for this is that changes in *ζ* are a mechanism for pre-emptive adaptation to changing conditions in plasma in normal physiology. This would allow RBCs to compensate for circadian changes in blood plasma composition, maintaining a constant level of electrostatic repulsion throughout the day. However, since this relies on two unconnected systems maintaining common rhythms, desynchronization in either amplitude or phase could potentially present dangers. It is notable that the period of the observed cycle where *ζ* is least polarised is between 0700 – 1200, the time window most associated with cardiovascular disease events such as heart attacks and strokes^38^. Further investigation on this topic, particularly considering in vivo measurements, may yield clinically beneficial information.

More speculatively, if the relationship between *V*_*m*_ and *ζ* occurs across other cell types, it may explain observed behaviours where cells exhibit changes in *V*_*m*_ when altering the way in which they interact with their environment. There is evidence to suggest this; it was recently shown that HeLa cells change *ζ* when subject to heat shock^36^, which our results suggest correlates to depolarisation due to loss of membrane integrity. Furthermore, several cell types exhibit changes in *V*_*m*_ when altering the way in which they interact with their environment. For example, as cancer cells increase in metastatic potential, their membrane potentials become more depolarised^6^, whilst membrane capacitance increases^8^; changes in *V*_*m*_ have been reported upon activation in macrophages^39^ and platelets^40^; ova of many species also change *V*_*m*_ immediately after fertilization^41^. Human sperm infertility has been associated both with low *ζ*^42^ and low membrane potential^43^, which our model suggests may be different measures of the same phenomenon. All of these cases correspond to cells which seek either to move into contact with other cells (platelets, macrophages) or else repel other cells (metastasising cancer cells, fertilised eggs), a process in which *ζ* may play a significant role.

In summary, we demonstrate an endogenously-generated electric field proportional to the membrane potential, and most likely caused by capacitive coupling between cytoplasm and extracellular medium, which manifests as changes in *ζ*-potential, low-frequency polarisation and surface conductance. Significantly, this shows RBCs can alter the way in which they interact with their surroundings by *V*_*m*_ rather than membrane composition, shedding new light on ion channel activity, cardiovascular disease, and drug action.

## Methods

### Goldman–Hodgkin–Katz (GHK) modelling

*V*_*m*_ was calculated using an adapted GHK equation. Extracellular ion concentrations were as below in ‘media’. Where an ion was absent from an extracellular solution, the concentration was set as 0 mM. Internal ion concentrations (mM) for RBCs^44^ were taken as: 140 K^+^, 11 Na^+^, 80 Cl^-^, and 0.006Ca^2+^. Relative permeabilities^45^ were taken as 100 for K^+^, 54 for Na^+^, 21 for Cl^−^ and 50 for Ca^2+^. Further details can be found in Table 3.

**Table 3:** Table of ion concentrations used in calculations.

Solution	NaCl		KCl		NaCl + KCl			CaCl_2_	
Ion	Na	Cl	K	Cl	Na	K	Cl	Ca	Cl_2_
[mM]o	145	145	145	145	72.5	72.5	145	0.97	0.97
[mM]i	11	80	140	80	11	140	80	0.0057	80
pX	54	21	100	21	54	100	21	50	21

The ion concentrations used in GHK modelling to determine the membrane potential in different ionic solutions.

### Blood cell preparation

Studies were conducted in accordance with the principles of the Declaration of Helsinki, with a favourable ethical opinion from the Research Ethics Committee at the University of Surrey. Participants in the study were screened for relevant self-reported health issues, including sleep disorders or excessive daytime sleepiness. Participants provided written, informed consent after having received a detailed explanation of the study procedures. RBCs were isolated from anticoagulant-treated whole blood by density gradient centrifugation layering a mixture of whole blood and PBS (1:3) on top of Histopaque-1077 (1.077 g/ml polysucrose solution) (Sigma-Aldrich, St Louis, MO) according to manufacturer’s instructions. RBCs were washed twice in PBS before resuspension in Krebs–Henseleit buffer (KHB, pH 7.4, 290 mOsm) (Sigma-Aldrich). These minimal medium conditions ensure the tiny fraction of nucleated cells detectable in RBC pellets immediately after centrifugation (~ 0.02%) undergo cell death in < 24 h and cannot influence RBC circadian rhythms, which is comparable to granulocyte depletion using anti-CD15 beads confirmed by gel zymography^10^. Circadian entrainment was achieved by 12:12 h 32 °C:37 °C temperature cycles over 48 h using a thermal cycler, and then used for experiments.

### Media and drug treatments

Isosmotic media were prepared for three experiments. In the first experiment, a physiological-strength ionic solution was prepared containing 145 mM NaCl and 7.5 mM KCl; this is referred to as the “100% solution” in the text. A second medium was prepared by diluting this 1:9 (final concentration 14.5 mM NaCl and 0.75 mM KCl) and a third, 1:99 using an isomotic solution containing 8.5% w/v sucrose and 0.5% w/v dextrose. These are referred to as the 10% and 1% solutions, respectively. For the second experiment, solutions were prepared of 145 mM KCl, 145 mM NaCl, 97 mM CaCl_2_ or a mixture containing 72.5 mM KCl and 72.5 mM NaCl. Cells were left to equilibrate with the media for 30 min prior to measurement, except where described in the text. For the third experiment, RBCs were suspended either in KHB as described above, or a low-conductivity “DEP medium” comprising an iso-osmotic sucrose-glucose solution adjusted to a conductivity of 0.043 S/m^10^ using phosphate-buffered saline (Labtech International, Heathfield, UK). In addition to untreated control cells, four treatments were used: neuraminidase (15ug/ml final concentration); valinomycin (30 nM final concentration) dissolved in DMSO; a combined treatment of both valinomycin and neuraminidase together; and a DMSO vehicle control (0.13%). All treatments were incubated for 30 min before measurement.

### Circadian rhythm analysis

RBCs were incubated at constant 37 °C for time course sampling, with a separate aliquot being removed from the cycler for analysis at each time point for each donor. The final transition to 37 °C was taken as t = 0 (or Zeitgeber time 0, ZT0). Measurements were taken every 3 h from ZT0 to ZT48. We also tested a single participant using blood taken via needlestick to the finger, approximately every 2 h for 24 h; a drop of blood was resuspended in 1 ml KHB and measured the ζ-potential immediately, with 3 measurements taken as before. Time course data were analysed using Prism 8 (Graphpad Software, La Jolla, CA). Curve fitting was used to fit a damped cosine in order to determine the circadian parameters baseline, period, amplitude and phase as described by Hirota et al.^46^; to determine if a circadian rhythm was present, a straight line fit (y = mx + c) was compared with a damped cosine + baseline fit (y = mx + c + amplitude x e(-kx) x cos(2.π.(X-phase)/period)) using the extra sum-of-squares F test in the compare models function of Prism 8, with the simpler model being preferred unless the p value was < 0.05.

### Measurement protocols

Data collection was performed using three methods in parallel, timed to ensure that measurements were as close in time to one another as possible (within 1 min).

#### Passive electrical properties

Passive electrical properties were measured using dielectrophoresis (DEP)^47^. The cell population was counted using a haemocytometer and adjusted to 1.15 × 10^6^ cells/ml (± 15%). 100 cell radii were measured using Image J software (National Institute of Health, Maryland, US) for use in the analysis. Cell suspensions were analysed using a 3DEP reader (Heathfield, UK)^22^. Cells were analysed for 60 s at five points per decade (10 kHz—20 MHz). Five technical repeats were performed for each of the four donors taken, and all data were averaged before fitting to a single-shell model^48^ using MATLAB (The Mathworks, USA) to determine *C*_*eff*_, *G*_*eff*_ and *σ*_*cyto*_. The model was adapted^23,24^ to include the contribution from the low-frequency double layer polarization observed in nanoparticles.

#### *ζ*-Potential

Ζ was measured using the dynamic light scattering method^49^. Samples were pipetted into disposable cuvettes and analysed using a Malvern Panalytical Zetasizer Nano ZS90 (Malvern, UK). Six technical repeats were taken per sample.

#### Membrane potential

*V*_*m*_ was determined using the CCCP (carbonylcyanide-m-chlorophenylhydrazone) method (Moersdorf et al.^19^), which allows rapid determination in a variety of media, including low ionic strengths where patch-clamp would be unable to form a suitable seal. To measure *V*_*m*_, 200 µl of packed cells were added to 4.8 mL of the test solution together with 20 µM (final concentration) of CCCP in a DMSO carrier. *V*_*m*_ was determined by monitoring of extracellular pH (pH_out_) in the presence of CCCP using a conventional pH electrode and calculated using the equation: *V*_*m*_ = 61.5 mV × (pH_in_ − pH_out_). Due to the high RBC buffer capacity, the intracellular pH (pH_in_) remains constant throughout the experiment. Final pH was determined as the pH in the solution after the cells were lysed by addition of 200 µl Triton X-100 (1% v/v) in 2 M NaCl solution.
